# Effect of 3D Printing Technology in Proximal Femoral Osteotomy in Children with Developmental Dysplasia of the Hip

**DOI:** 10.1155/2022/1291996

**Published:** 2022-02-22

**Authors:** Jin Cao, Chao Gao, Jing Hua Sun, Hua Jiang Zheng, Huan Ye Zhu, Zhao Ping Zhong, Long Zhou

**Affiliations:** ^1^Department of Orthopedics, Ningbo Sixth Hospital, 1059 Zhongshan East Rd, Ningbo, Zhejiang 315000, China; ^2^Department of Pathology, Ningbo Diagnostic Pathology Center, Ningbo, Zhejiang 315000, China

## Abstract

**Objective:**

To investigate the effect and safety of 3D printing technology in proximal femoral osteotomy in children with developmental dysplasia of the hip.

**Methods:**

40 cases of children with developmental dysplasia of the hip treated by pelvic osteotomy combined with proximal femoral osteotomy at Ningbo No. 6 Hospital from January 2017 to December 2019 were retrieved and retrospectively analyzed. Among them, 20 cases received preoperative measurement and design assisted by 3D printing technology (the 3D printing group), and 20 cases received conventional preoperative measurement and design (the conventional group).

**Results:**

All patients were followed up for an average of 25 (12~36) months. During the follow-up, there were no complications such as infection, fracture of internal fixation, or malunion of osteotomy. Compared with the conventional group, the 3D printing group had a shorter operation time, less intraoperative blood loss, and fewer intraoperative X-ray fluoroscopies (all *p* < 0.05). In the last follow-up, the clinical efficacy was evaluated by the McKay standard: in the 3D printing group, 14 cases were excellent, 5 cases were good, and 1 case was fair. In the conventional group, 10 cases were excellent, 9 cases were good, and 1 case was fair (*Z* = −0.382, *p* > 0.05).

**Conclusion:**

Preoperative 3D printing of bilateral femur and other large physical models is accurate, which is ideal for the development of individual preoperative planning. Proximal femoral osteotomy using preoperative measurements and simulated surgical data improves the safety of the operation.

## 1. Introduction

Developmental dysplasia of the hip (DDH) is a common hip deformity in children, and pelvic osteotomy combined with proximal femoral rotation, varus, and shortening osteotomy is a commonly used surgical treatment [1, 2]. Accurate osteotomy during operation, an important factor, is directly related to the therapeutic effect and accurate measurement before operation. Previous surgeons often evaluate the degree of proximal femoral deformity according to preoperative X-ray and CT measurement results and perform proximal femoral osteotomy according to personal experience during the operation. However, there are huge errors in preoperative measurement and intraoperative operation, so it is necessary to find a more accurate method [3]. The application of 3D printing technology in the orthopedics provides the possibility for individualized and precise treatment of patients and can be used for preoperative measurement and surgical planning of patients with developmental hip dysplasia, thus effectively reducing the errors caused by surgeon's personal experience. In this study, based on 3D printing technology, a bilateral equal-large femur model of the patient was printed, the femoral neck anteversion and other parameters were measured, the intraoperative operation was simulated, and a more accurate and convenient osteotomy method for the proximal femur was explored.

## 2. Materials and Methods

### 2.1. Design

A retrospective design was used. This study was approved by the Institutional Review Board of Ningbo No. 6 Hospital. Written informed consent was obtained from all the patients.

### 2.2. Diagnosis Criteria

The diagnosis criteria were as follows: (1) Ortolani test positive; (2) Barlow test positive; (3) dislocatable hip on dynamic ultrasound; (4) asymmetry in abduction ≥ 20°; (5) *α* angle < 45° on ultrasound; (6) abduction limited to 45°; (7) leg-length discrepancy; and (8) any asymmetry of hip abduction.

### 2.3. Case Inclusion and Exclusion Criteria

**Figure 1:**
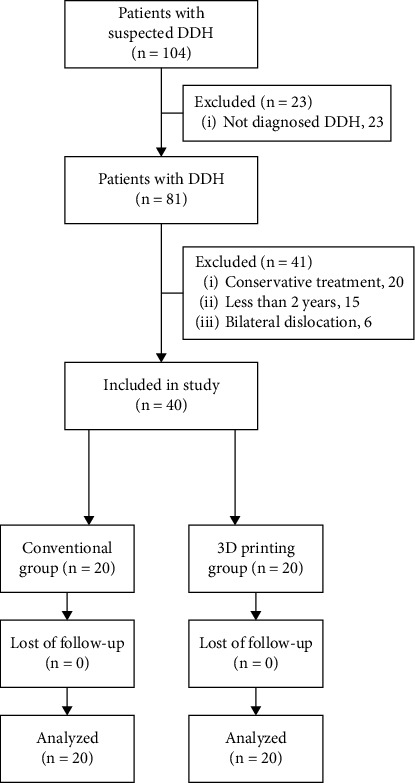
Case flow chart.

The inclusion criteria were as follows: (1) confirmed diagnosis of DDH; (2) aged 2 years or over; and (3) received surgical treatment for DDH. The exclusion criteria were as follows: (1) bilateral dislocation or (2) complex neuromuscular diseases. A total of 104 patients were retrieved and screened. Forty of them met the inclusion criteria. Follow-up was performed on an outpatient basis. There were no lost patients. 20 cases received operation assisted with 3D printing technology (Group A), and the other 20 cases received conventional operation (Group B) (Figure 1).

### 2.4. General Information

This study included 40 children with developmental dysplasia of the hip treated with pelvic osteotomy combined with proximal femoral osteotomy at Ningbo No. 6 Hospital from January 2017 to December 2019 (Table 1). In the 3D printing group, 15 cases were male and 5 cases were female (age: 8~14 years old; average age: 9.1 years old; 12 hips on the left and 8 hips on the right; Tonnis classification: Grade 1 included 3 hips, Grade 2 included 7 hips, Grade 3 included 6 hips, and Grade 4 included 4 hip); 14 patients were treated for the first time, and 6 patients were treated after failure of nonsurgical treatment. In the conventional group, 13 cases were male and 7 cases were female (age: 6~13 years old; average age: 9.7 years old). There were 14 left hips and 6 right hips, Tonnis classification: Grade 1 included 2 hips, Grade 2 included 6 hips, Grade 3 included 8 hips, and Grade 4 included 4 hips; 13 patients received surgery for the first time, and 7 patients received surgery after failure of nonsurgical treatment.

### 2.5. Treatment

#### 2.5.1. Preoperative Planning

For Group A, preoperative anteroposterior and abducent internal rotation X-ray films of the pelvis were taken, and 64-slice spiral CT scanning of the pelvis and bilateral femur was performed. The original DICOM data obtained were imported into medical image segmentation software (Mimics Innovation Suite v15.0, Materialise, Belgium), and the three-dimensional models of the pelvis and bilateral femur were reconstructed by the software. The physical models of the pelvis and bilateral femur were produced by a 3D printer (3d medical image processing software 4.1.3, Zhejiang Derda Medical Technology Co., Ltd, Ningbo). The femoral neck anteversion was measured by the physical model of the femur, and the proximal femur simulated osteotomy was performed with the healthy side as the reference to recover the abnormal anteangle and neck-shaft angle of the proximal femur, and then, the appropriate steel plate was selected for fixation. Those with the femoral neck anteversion > 40° were rotated to restore the normal angle, which was corrected to 15°~25° according to different ages. If the femoral neck anteversion was less than 40°, it could not be corrected. Varus osteotomy was performed for patients with increased neck-shaft angle and corrected to about 130°. The osteotomy site, rotation distance, varus angle, and shortening distance of the femur were measured and recorded.

For Group B, preoperative anteroposterior and abducent internal rotation X-ray films of the pelvis were taken, and 64-row spiral CT scans were performed on the bilateral hip joints and bilateral femurs of the patients. Images of the affected hip and bilateral femur were reconstructed in the computer using PACS software (V4.9.2389.582_20210617, Tomorrow Medical Network Technology Co., Ltd, Ningbo). Various parameters of the proximal femur were measured by X-ray films and traditional CT measurement methods [4, 5].

#### 2.5.2. Surgical Procedures

After successful general anesthesia, the patient was placed in a supine position and the affected buttocks were padded high. Femoral lateral and longitudinal incisions were performed from the top of the great trochanter. After cutting the vastus lateralis and step by step separation, the proximal femur was exposed to the edge of the great trochanter. According to preoperative simulation measurements of bone, the varus rotating and shortening osteotomy was completed under the femoral trochanter. After achieving correction, proximal humerus plates (PHP) were used to fix the femur. Pelvic osteotomy was performed routinely, and the affected limb was fixed with a hip herringbone cast. The cast was replaced 6 weeks later, and the cast was removed at 12 weeks after surgery and weight-bearing began. The fixation was removed after the femoral osteotomy was completely healed.

### 2.6. Measures and Data Collection

The preoperative general conditions of the two groups were recorded. Operation time, intraoperative fluoroscopy times, and intraoperative blood loss were compared between the two groups. Hip function was assessed on the MacKay hip scale (Table 2) at 4 weeks postoperatively.

### 2.7. Statistical Analysis

The operative time, intraoperative blood loss, number of intraoperative X-ray fluoroscopies, and clinical efficacy were compared between the two groups. Clinical efficacy was assessed by the McKay criteria [6]. SPSS 23.0 statistical software was used to process data. Measurement data were expressed as mean ± standard deviation ($\bar{x}\pm s$). Two independent-samples *t*-test was used for comparison of operative time, intraoperative blood loss, and number of intraoperative X-rays between groups. The rank sum test was used to compare the grade data. A 2-sided *p* value less than 0.05 was considered as significant difference.

## 3. Results

**Figure 2:**
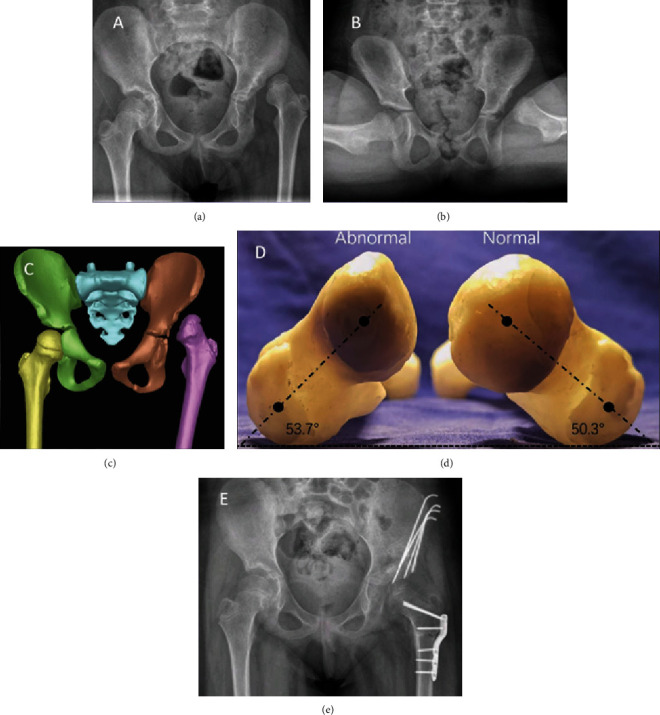
The patient was a 12-year-old female with left DDH and progressive left hip pain for 1 year. She underwent left pelvic osteotomy combined with proximal femoral shortening and varus rotating osteotomy. (a, b) Preoperative radiography of the pelvis showed dislocation of the left hip joint. (c) The pelvis 3D model showed dislocation of the left hip joint. (d) Measuring the femoral neck anteversion by the femoral model. (e) Anteroposterior radiography of the pelvis after 3-month postoperation, indicating the reduction of the hip joint.

All 40 patients were successfully followed up for an average of 25 (12-36) months. During the follow-up, there were no complications such as infection, fracture of internal fixation and malunion of bone cutting place. The operation time of the 3D printing group was (23.90 ± 2.88) minutes, and that of the conventional group was (50.70 ± 4.22) minutes (t =16.585, p <0.05). The intraoperative blood loss was 37.50 ± 4.09 ml in the 3D printing group and 58.80 ± 4.10 ml in the conventional group (*t* = 11.626, *p* < 0.05). The number of intraoperative X-ray fluoroscopies was 4.40 ± 0.84 times in the 3D printing group and 6.80 ± 1.03 times in the conventional group (*t* = 5.692, *p* < 0.05, Table 3). In the last follow-up, the clinical efficacy was evaluated by McKay standard: 5 cases in the 3D printing group were excellent, 4 cases were good, and 1 case was fair. In the conventional group, 6 cases were excellent, 3 cases were good, and 1 case was fair. There was no statistically significant difference between groups (*Z* = −0.382, *p* > 0.05). A typical case is shown in Figure 2.

## 4. Discussion

Pediatric patients show impressive correction and regeneration capabilities [8, 9]. Children under 2 years with DDH only need conservative treatment. For children with DDH older than 2 years, surgical treatment is the better option, and the purpose of treatment is to achieve the reduction of concentric circles of the acetabulum, so as to restore the normal anatomical structure of the hip joint and the optimal joint function, which is also the key to the postoperative evaluation of the treatment effect [10, 11]. Surgical procedures include a pelvic osteotomy and a proximal femoral osteotomy, which often involves derotation, varus, and shortening osteotomy. Since the increase of femoral neck anteversion is one of the important pathological changes in developmental hip dysplasia, rotatory osteotomy of the proximal femur is often used to correct the increased angle in children with developmental hip dysplasia [12–14], so as to finally restore the approximate normal anatomical structure of the proximal femur. But whether or not to rotate and how to rotate precisely should be evaluated preoperatively. Because there is no accurate method to measure the femoral neck anteversion during routine surgery, the best angle of rotation is determined by the surgeons according to their experience during the operation, which obviously lacks accuracy and consistency, affects the surgical results, and limits the development of surgical techniques. Many pediatric orthopedic surgeons have the trouble: knowing the angle is bigger in patients with developmental dysplasia of hip before operation, but cannot preoperatively determine how much bigger the angle should be and whether it must be corrected during the operation. Without a quantitative standard, for inexperienced surgeons, it will inevitably affect the outcome of the operation. Therefore, a more accurate preoperative measurement method is needed clinically to assist in accurate and rapid completion of proximal femoral osteotomy in order to obtain better surgical efficacy.

3D printing is a new technology developed in the 21st century and has been rapidly applied in the field of medicine [15–17]. 3D printing technology deals with femoral imaging data by software and makes equal large physical models with the help of a 3D printer. The pathological characteristics of the proximal femur of the affected side can be intuitively understood by the model, and parameters such as the femoral neck anteversion can be accurately measured. Surgeons can use these data to make precise surgical plans. 3D printing technology can also help young surgeons better understand proximal femoral osteotomy and shorten the learning curve of the surgery. At the same time, various parameters can be measured and simulated surgeries can be performed on the femur model of the patient, so as to develop personalized surgical plan and make the osteotomy surgery more accurate. In the 3D printing group of this study, CT scans were performed on the full length of bilateral femurs of 10 children with DDH. The data were processed by Mimics software, and an equal large physical model was made to accurately measure the femoral neck anteversion. Secondly, for each child, the proximal femoral osteotomy was planned for virtual surgery. The rotation distance and plate placement were recorded according to the results of the simulated surgery, and the precise osteotomy was completed according to the simulated surgical data during the operation. In this study, there was no less rotation, overrotation, high osteotomy position, or low osteotomy position in the 3D printing group. So, we suggest that 3D printing technology can assist in the completion of proximal femoral osteotomy, reduce the complications of the operation, and improve the surgical efficacy.

Routine proximal femoral osteotomy mostly depends on the experience of the operator. Repeated X-ray fluoroscopy during the operation to achieve the best reduction effect will obviously prolong the operation time, increase the amount of intraoperative bleeding and X-ray, and even affect the surgical results. The 3D printing model can effectively assist the accurate and rapid completion of osteotomy, reduce the operative time and intraoperative blood loss, and reduce the number of intraoperative X-ray of patients. The operative time, intraoperative bleeding, and X-ray exposure times of 10 cases in this group were better than those of traditional surgical methods. The advantages of 3D printing technology in proximal femoral osteotomy of children with DDH are as follows: (1) proficient in surgical procedures to improve the accuracy of osteotomy; (2) it can shorten the operation time and reduce the amount of intraoperative bleeding and the number of intraoperative fluoroscopies; and (3) preoperative simulation surgery can reduce the difficulty of operation, which prepares young doctors better and faster to master the technical points of proximal femoral osteotomy. In contrast to the above advantages, 3D printing technology also has its limitations: (1) additional costs for 3D model printing and (2) the accuracy of osteotomy is related to intraoperative exposure. Because there is no muscle attachment in the 3D model, the simulated operation field is wider, and more soft tissues need to be removed in order to have better exposure during the operation.

In conclusion, the application of 3D printing technology in proximal femoral osteotomy in children with DDH can achieve satisfactory results. Preoperative 3D printing of bilateral equal large femoral models for accurate measurement and surgical simulation is conducive to the individual preoperative plans. Proximal femoral osteotomy with preoperative measurements and simulated surgical data improves the safety and accuracy of the operation and reduces the difficulty of the operation.

## Figures and Tables

**Table 1:** General information.

	Gender		Side		Age	Tonnis classification				Primary surgery	
	M	F	L	R		I	II	III	IV	Yes	No
3D group (*N* = 20)	15	5	12	8	9.1 ± 2.1	3	7	6	4	14	6
Conventional group (*N* = 20)	13	7	14	6	9.7 ± 2.1	2	6	8	4	13	7
*p* value	0.4902		0.5073		0.3802	0.9042				0.7357	

Chi-square test.

**Table 2:** McKay's criteria for clinical evaluation [6, 7].

Grade	Criteria
Excellent	Stable, painless hip; no limp; negative Trendelenburg sign; full range of motion
Good	Stable, painless hip; slight limp; slight decrease in range of motion
Fair	Stable, painless hip; limp; positive Trendelenburg sign; and limited range of motion or a combination of these
Poor	Unstable or painful hip or both; positive Trendelenburg sign

**Table 3:** Comparison between the two groups in the time, blood loss, and radiation times of proximal femoral osteotomy ($\bar{x}\pm s$).

Group	Cases	Time (min)	Blood loss (ml)	Time of X-ray (times)
3D printing group	20	23.90 ± 2.88	37.50 ± 4.09	4.40 ± 0.84
Conventional group	20	50.70 ± 4.22	58.80 ± 4.10	6.80 ± 1.03
*t* value		16.585	11.626	5.692
*p* value		<0.0001	<0.0001	<0.0001

*t*-test.

## Data Availability

All data included in this study are available upon request by contact with the corresponding author.
